# Differential expression of interferon-γ and chemokine genes distinguishes Rasmussen encephalitis from cortical dysplasia and provides evidence for an early Th1 immune response

**DOI:** 10.1186/1742-2094-10-56

**Published:** 2013-05-02

**Authors:** Geoffrey C Owens, My N Huynh, Julia W Chang, David L McArthur, Michelle J Hickey, Harry V Vinters, Gary W Mathern, Carol A Kruse

**Affiliations:** 1 Department of Neurosurgery, David Geffen School of Medicine, University of California, Los Angeles, CA, USA; 2 Department of Pathology and Laboratory Medicine, David Geffen School of Medicine, University of California, Los Angeles, CA, USA; 3 Department of Neurology, David Geffen School of Medicine, University of California, Los Angeles, CA, USA; 4 Intellectual and Developmental Disabilities Research Center, David Geffen School of Medicine, University of California, Los Angeles, CA, USA; 5 Mattel Children’s Hospital, David Geffen School of Medicine, University of California, Los Angeles, CA, USA; 6 Brain Research Institute, David Geffen School of Medicine, University of California, Los Angeles, CA, USA; 7 UCLA Neurosurgery, Gonda Center, Room 1554, Box 951761, Los Angeles, CA, 90095, USA

**Keywords:** Inflammation, Rasmussen encephalitis, Cortical dysplasia, Epilepsy, Gene expression, T cells, Chemokines

## Abstract

**Background:**

Rasmussen encephalitis (RE) is a rare complex inflammatory disease, primarily seen in young children, that is characterized by severe partial seizures and brain atrophy. Surgery is currently the only effective treatment option. To identify genes specifically associated with the immunopathology in RE, RNA transcripts of genes involved in inflammation and autoimmunity were measured in brain tissue from RE surgeries and compared with those in surgical specimens of cortical dysplasia (CD), a major cause of intractable pediatric epilepsy.

**Methods:**

Quantitative polymerase chain reactions measured the relative expression of 84 genes related to inflammation and autoimmunity in 12 RE specimens and in the reference group of 12 CD surgical specimens. Data were analyzed by consensus clustering using the entire dataset, and by pairwise comparison of gene expression levels between the RE and CD cohorts using the Harrell-Davis distribution-free quantile estimator method.

**Results:**

Consensus clustering identified six RE cases that were clearly distinguished from the CD cases and from other RE cases. Pairwise comparison showed that seven mRNAs encoding interferon-γ, CCL5, CCL22, CCL23, CXCL9, CXCL10, and Fas ligand were higher in the RE specimens compared with the CD specimens, whereas the mRNA encoding hypoxanthine-guanine phosphoribosyltransferase was reduced. Interferon-γ, CXCL5, CXCL9 and CXCL10 mRNA levels negatively correlated with time from seizure onset to surgery (*P* <0.05), whereas CCL23 and Fas ligand transcript levels positively correlated with the degree of tissue destruction and inflammation, respectively (*P* <0.05), as determined from magnetic resonance imaging (MRI) T2 and FLAIR images. Accumulation of CD4^+^ lymphocytes in leptomeninges and perivascular spaces was a prominent feature in RE specimens resected within a year of seizure onset.

**Conclusions:**

Active disease is characterized by a Th1 immune response that appears to involve both CD8^+^ and CD4^+^ T cells. Our findings suggest therapeutic intervention targeting specific chemokine/chemokine receptors may be useful in early stage RE.

## Background

Rasmussen encephalitis (RE) is an inflammatory neurodegenerative disease primarily seen in young children. It is clinically characterized by intense focal and generalized seizures with inflammation almost invariably confined to one cerebral hemisphere [1-3]. Seizure frequency may decrease over time, but patients are left with unilateral hemiparesis and significant cognitive deficits [4,5]. Histopathologic examination of RE brain tissue, usually after years of seizures, reveals the presence of activated microglia and T lymphocytes [6-8]. CD8^+^ T cells containing granzymes are observed in close apposition to neurons and astrocytes, but not oligodendrocytes [6,9]. Auto-antibodies against the glutamate receptor GluR3, once considered a possible cause of RE [10], are not present in all RE cases and are not specific to the disease [11-13]. Circulating antibodies to other neuronal proteins are found in other RE cases [14-16], leading to the view that a humoral immune response may be a secondary event to T cell immunity [6,17]. Thus, RE resembles an autoimmune disease although the histopathology is also consistent with an immune response to a virus or other infectious agent [1,17]. Cytomegalovirus, herpes simplex virus, and Epstein Barr virus sequences have been detected in some RE brain specimens, but have not been reproducibly identified in all cases [18-22]. Neither autoimmunity nor infection can easily explain the unilateral hemispheric involvement.

To gain further insight into the immunopathology of RE, we measured the relative expression of 84 mRNA transcripts associated with inflammation and autoimmunity in brain tissue from 12 RE and 12 cortical dysplasia (CD) epilepsy patients. The CD cases constituted a reference group against which to compare gene expression levels. Inflammation is associated with CD, but is less severe than in RE, and T cell involvement is limited [23,24]. Quantitative differences in transcript levels between RE and CD specimens were found for several genes involved in the activation and recruitment of effector T cells. The highest levels of expression were found in early stage RE cases.

## Methods

### Cohort recruitment and clinical variables

Under University of California, Los Angeles, (UCLA) Institutional Review Board (IRB) approval, brain tissue was collected at surgery as part of UCLA’s Pediatric Epilepsy Surgery program. Informed consent to use the surgically resected tissue for research was obtained through the parents or legal guardians. The 12 RE and 12 CD cases used in the study were selected with similar ages at surgery. Seven CD cases were classified as CDI, and five as CDII [25]. The clinical protocols for patient evaluation and surgical procedures for collection and processing of cortical specimens have been previously published [26,27]. Clinical variables included, age at seizure onset, age at surgery, and disease progression (age at seizure onset to age at surgery). Magnetic resonance imaging (MRI) scans were assessed by one investigator (GWM). A semiquantitative score was assigned to the T2 and FLAIR signal changes (0 = none, 1 = slight; 2 = mild; 3 = moderate; 4 = extensive) to estimate the degree of tissue destruction and inflammation, respectively.

### Real-time PCR and analysis

Total RNA was purified from flash frozen blocks of involved tissue consisting of mostly cortical gray matter (approximately 50 mg) using Trizol™ (Life Technologies, Carlsbad, CA, USA) and reverse transcribed (Qiagen, Valencia, CA, USA). PCR reactions were carried out in an ABI 7300 thermocycler using SYBR™ green chemistry (SABiosciences, Valencia, CA, USA). The 96-well format qPCR array contained primers for 84 genes of interest and 5 reference genes (SABiosciences inflammation and autoimmunity qPCR array, cat no. PAHS-0077Z). Standard cycling parameters were used as follows: 1 cycle: 95°C 10 min, 40 cycles: 95°C 15 sec, 60°C 1 min. As part of the cycling program, all PCR products were thermally denatured and dissociation curves were obtained. Two of the primer sets resulted in dissociation curves with multiple peaks (CCL16 and CCL24) and were eliminated from the analysis. To calculate relative transcript levels, baseline-subtracted fluorescence values per cycle for each primer set were entered into LinRegPCR [28] and PCR efficiencies (E) and Ct values were determined. The relative expression of each gene (X_0_) [29] in each array was calculated from:

$$X_{0}=E^{-\mathrm{Ct}}$$

NormalFinder [30] was used to identify the least variable housekeeping gene across the 24 arrays. *ACTB* encoding β-actin was found to be the most stable reference gene, and was used to normalize all of the data including the four other reference genes (*HPRT1*, *RPL13A*, *GAPDH*, *B2M*). Samples were grouped by consensus clustering [31] using the non-negative matrix factorization algorithm [32] found in the GENE-E bioinformatics package (http://www.broadinstitute.org). Statistical analyses utilized R-project programs (http://www.r-project.org). Plots were generated by the Deducer graphical user interface and exported into CorelDRAW X6 (Corel Corporation, Ottawa, ON, Canada). Significant differences (*P* <0.05) in gene expression between RE and CD cases were determined by pairwise comparison of the distribution of transcript levels for each gene using Harrell-Davis quantile estimators [33,34].

### Immunocytochemistry and image analysis

Paraffin-embedded blocks of involved tissue were serially sectioned (5 μm), deparaffinized, and microwaved for 20 minutes in buffered citrate (10 mM, pH 6.0) for antigen retrieval. After one hour in blocking solution (Impress Kit, Vector Laboratories, Burlingame, CA, USA) sections were incubated overnight at 4°C with rabbit anti-human CD4 (1:250, Novus Biologicals, Littleton, CO, USA) or mouse anti-human CD8 (1:100, Dako, Carpinteria, CA, USA). Sections were immunostained for one hour at 25°C with peroxidase-conjugated anti-rabbit or anti-mouse secondary antibodies (Impress Kit, Vector Laboratories) followed by incubation with 3,3′-diaminobenzidine (DAB) substrate (MP Biomedicals, Santa Ana, CA, USA), then counterstained with hematoxylin. Sections of tonsil tissue were used as positive controls, and omission of primary antibodies served as negative controls. Images of entire sections were acquired with an Aperio ScanScope XT scanner (Aperio, Vista, CA, USA) and transferred to CorelDRAWX6. Strong DAB staining of CD4 and CD8 immunoreactive cells was quantified using the positive pixel count algorithm, part of the Aperio ImageScope software package.

### Western blotting

Blocks of flash frozen involved tissue were homogenized in RIPA buffer containing protease and phosphatase inhibitors (Sigma-Aldrich, St. Louis, MO, USA). Lysates were separated on precast 10% polyacrylamide gels (Biorad, Hercules, CA, USA) and transferred to PVDF membranes (Biorad). Prestained molecular standards were used (Biorad). The membrane was blocked in Tris-buffered saline (pH 7.4) containing 5% nonfat dried milk and 0.1% Tween™ 20, and probed with a monoclonal antibody to hypoxanthine-guanine phosphoribosyltransferase (anti-HPRT 1:1000, Proteintech Group Inc., Chicago, IL, USA). Proteins were visualized with a secondary antibody conjugated to horseradish peroxidase (1:2500, Jackson ImmunoResearch Laboratories, West Grove, PA, USA) using a chemiluminescent substrate (Thermo-Scientific, Waltham, MA, USA). The blot was stripped and re-probed with a monoclonal antibody to glyceraldehyde 3-phosphate dehydrogenase (anti-GAPDH 1:1000, Stressgen, Victoria, BC, Canada). X-ray films were scanned and processed (background subtraction and enhancement using default settings) in Image J, and exported to CorelDRAWX6.

## Results

### Rasmussen encephalitis and cortical dysplasia patient cohorts

**Figure 1:**
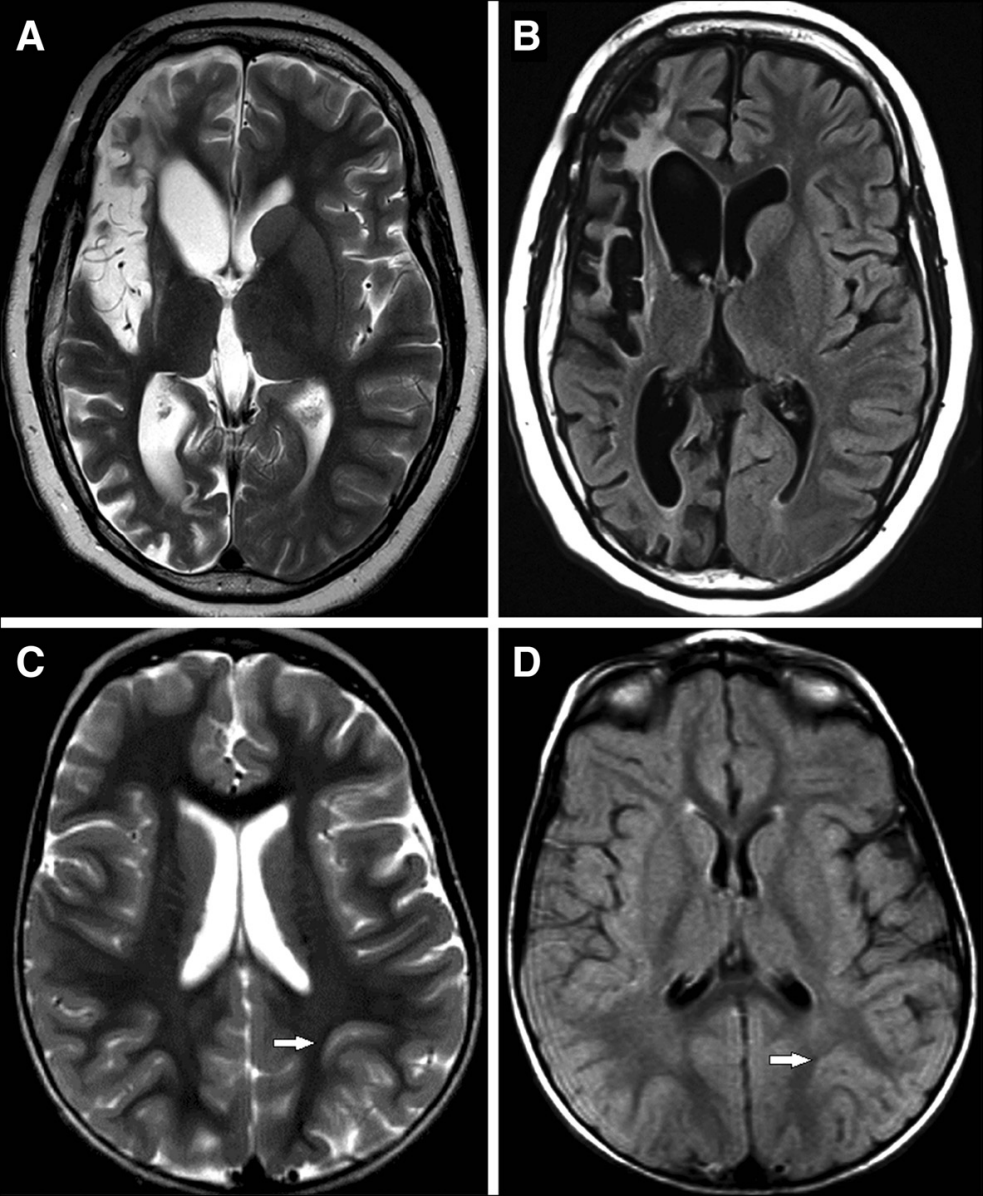
**Examples of magnetic resonance imaging (MRI) scans from patients with Rasmussen encephalitis (RE) or cortical dysplasia (CD).** Scans were semiquantitatively assessed for T2 (**A** and **C**) and FLAIR (**B** and **D**) signal changes. The RE case (**A** and **B**) was a 13 year old with a five-year history of seizures originating from the right hemisphere. The T2 image was scored severe (4) for tissue destruction (**A**), and moderate (3) for amount of FLAIR changes (**B**). The patient with CD (**C** and **D**) had abnormality localized to the left parietal area (arrows). The MRI scans were scored zero (no increased signal changes) for T2 (**C**) and FLAIR changes (**D**) despite the abnormal anatomy.

Table 1 summarizes clinical variables for the RE (n = 12) and CD (n = 12) cohorts selected for study. Patient data associated with each specimen are provided in Table 2. The age range at surgery was similar, whereas seizure onset occurred at an earlier age in the CD compared with the RE cohort, thus time from seizure onset to surgery also differed. Other clinical parameters were not significantly different, except for the extent of tissue destruction and inflammation measured by changes in the T2-weighted and FLAIR MRI scans, respectively. These parameters were scored semiquantitatively and reflect more injury/inflammation in RE compared with CD cases (Tables 1 and 2). Figure 1 shows examples of graded MRI scans from an RE (A and B) and CD patient (C and D).

**Table 1:** Summary of clinical variables for Rasmussen encephalitis (RE) and cortical dysplasia (CD) cohorts

**Clinical variable**	**RE (n = 12)**	**CD (n = 12)**	***P *value**
Age at surgery (years)	9.9 ± 3.1	9.2 ± 3.6	*P* = 0.621^a^
Age at seizure onset (years)	6.4 ± 3.1	2.8 ± 2.4	***P *= 0.005**^**a**^
Time from onset to surgery (years)	3.5 ± 3.5	6.5 ± 3.3	***P *= 0.047**^**a**^
Number of AED pre-surgery	3 ± 1	3 ± 1	*P* = 0.248^a^
Side (Left/Right)	4/8	6/6	*P* = 0.408^b^
Gender (Female/Male)	5/7	8/4	*P* = 0.219^b^
T2 Index	2.5	0	***P*= 0.016**^**c**^
FLAIR Index	2	1	***P *= 0.003**^**c**^

Mean ± SD or median values are shown. *P* values calculated as ^a^t-test.

^b^Chi square or ^c^Mann-Whitney. AED, antiepileptic drugs; FLAIR, fluid attenuated inversion recovery.

**Table 2:** **Clinical variables associated with Rasmussen encephalitis and** c**ortical dysplasia patient specimens**

**Specimen**	**Gender**	**Age at onset (years)**	**Age at surgery (years)**	**Onset to surgery (years)**	**Number of AEDs at surgery**	**Etiology**	**Affected hemisphere**	**Operation**	**T2**^**a**^	**FLAIR**^**a**^
RE1	M	4	4.75	0.75	2	RE	Right	Hemispherectomy	1	4
RE2	F	6.7	7.5	0.8	5	RE	Right	Hemispherectomy	3	1
RE3	F	4.5	10	5.5	4	RE	Right	Hemispherectomy	2	1
RE4	M	2.5	10	7.5	3	RE	Left	Hemispherectomy	1	1
RE5	M	9.25	10.5	1.25	3	RE	Left	Hemispherectomy	3	2
RE6	F	8	13	5	0	RE	Right	Hemispherectomy	4	3
RE7	M	12	13.5	1.5	4	RE	Left	Hemispherectomy	1	2
RE8	M	8	10.1	2.1	2	RE	Right	Hemispherectomy	2	1
RE9	F	4.1	6.25	2.15	4	RE	Right	Hemispherectomy	3	2
RE10	F	10	12.2	2.2	4	RE	Right	Hemispherectomy	3	3
RE11	M	2	14.4	12.2	4	RE	Left	Hemispherectomy	3	2
RE12	M	5.3	6.3	1	2	RE	Right	Hemispherectomy	1	1
CD1	F	5	14.2	9.2	2	CD1	Right	Temporal	1	1
CD2	F	0.75	3.2	2.45	2	CD1	Left	Frontal-Parietal	0	0
CD3	F	1.5	10.2	8.7	3	CD1/HS	Left	Temporal	0	1
CD4	F	3	6.5	3.5	1	CD1	Right	Temporal	1	1
CD5	F	1.4	9	7.6	4	CD	Right	Frontal	1	1
CD6	F	9	12.3	3.3	3	CD2A	Left	Temporal	1	1
CD7	F	3	8.5	5.5	2	CD2A	Right	Temporal	0	0
CD8	M	2.3	14.5	12.2	3	CD1	Left	Temporal-Parietal	0	1
CD9	M	0.1	5.2	5.1	2	CD1	Left	Parietal	0	1
CD10	F	0.75	9.3	8.55	4	CD1	Right	Hemispherectomy	0	0
CD11	M	3	12	9	3	CD2B	Left	Parietal	2	1
CD12	M	3.5	5.25	1.75	1	CD1	Right	Hemispherectomy	0	0

^a^Evaluation of T2 and FLAIR signal changes on scales of 0 to 4.

AED, antiepileptic drugs; CD, cortical dysplasia; FLAIR, fluid attenuated inversion recovery; HS, hippocampal sclerosis; RE, Rasmussen encephalitis.

### A subset of inflammatory genes distinguishes Rasmussen encephalitis from cortical dysplasia

We obtained normalized expression data for 86 genes (82 inflammatory and 4 housekeeping genes). Transcripts of 71 genes were detected in every specimen, confirming that inflammatory genes are expressed in both RE and CD [24]. Thirteen of the remaining genes were expressed in at least six RE specimens, and were retained for analysis. IL23R and IL1F10 transcripts were detected in only three of the RE specimens and were eliminated, leaving a total of 84 genes. IL23R transcripts were expressed in four of the CD cases, and IL1F10 expression was not detected in any of the CD cases.

**Figure 2:**
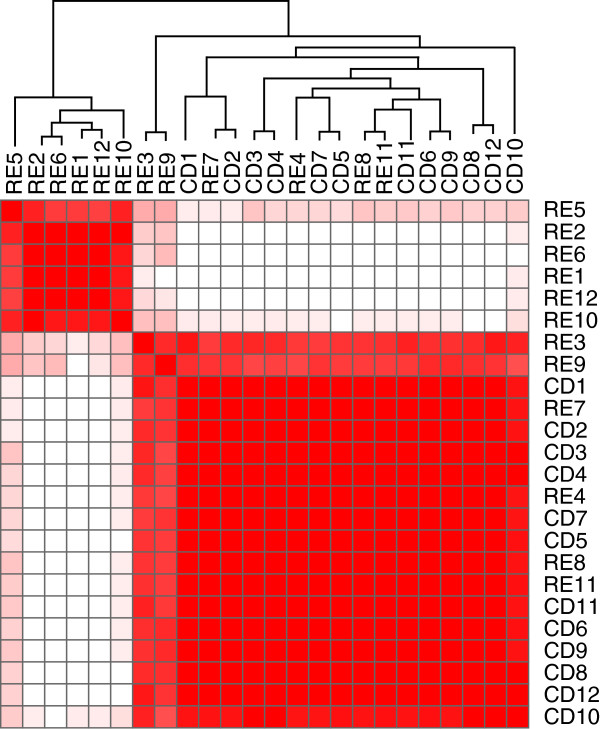
**Consensus clustering applied to qPCR array expression data from Rasmussen encephalitis (RE) and cortical dysplasia (CD) specimens.** Non-negative matrix factorization was used to determine whether the mRNA expression data could distinguish between RE and CD. With k = 2 clusters, six of the RE cohort were clearly predicted. Two RE cases partially segregated, and the remaining four could not be differentiated from the CD cohort. Heat map is scaled from high (red) to low (white) correlation between samples.

We employed a consensus clustering method to organize the blinded samples into two groups. Half of the RE cases (RE1, 2, 5, 6, 10, 12) segregated from the 12 CD specimens, two (RE3, 9) partially segregated, and the remaining four (RE4, 7, 8, 11) could not be distinguished from the CD cases (Figure 2). Thus, 6 of 12 RE cases could be clearly identified based solely on the array data. Sample heterogeneity may partly explain why not all of the RE cases clustered. On the other hand, the six clustered RE cases may indicate differences related to clinical variables such as disease progression [35].

**Figure 3:**
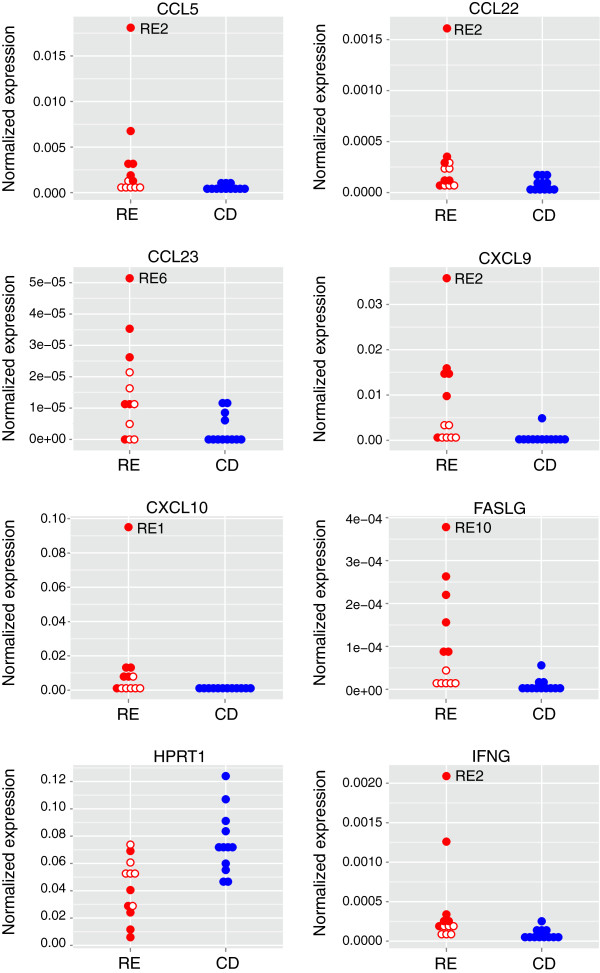
**Expression levels of genes that were significantly different between Rasmussen encephalitis (RE) and cortical dysplasia specimens.** Stacked dot plots of normalized expression values for each sample are shown. Solid circles identify the six RE specimens separable by consensus clustering (Figure 2); open circles identify the remaining RE cases. The RE sample with the highest inflammatory gene transcript level is indicated.

**Figure 4:**
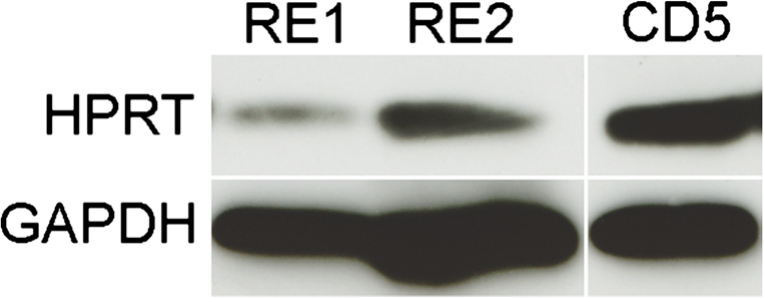
**Hypoxanthine-guanine phosphoribosyltransferase (HPRT) protein levels differ between Rasmussen encephalitis (RE) and cortical dysplasia (CD) specimens.** Western blot showing lower amounts of HPRT in two RE samples (RE1 and RE2) compared with a reference CD specimen (CD5). Glyceraldehyde-3-phosphate dehydrogenase (GAPDH) was used as a loading control.

Although the clustering analysis revealed variability within the RE cohort, we proceeded to examine whether the expression of particular genes differed between the RE and CD cohorts. The distribution of transcript levels of 40 genes in the RE set and 36 genes in the CD set did not satisfy the Shapiro-Wilk normality test (*P* <0.05); >85% of these genes showed *P* values of <0.01. Therefore pairwise comparisons were carried out using the Harrell-Davis distribution-free quantile estimator method. This generates robust analyses that are well-protected from violations of normality. The analysis uses an adjusted critical value for determining significance that controls for multiple comparisons. Distributions were divided into deciles. Transcript levels of eight genes were significantly different between the RE and CD samples across all quantiles (*P* <0.05; [see Additional file 1: Table S1]). Seven genes were expressed at higher levels in the RE samples; whereas hypoxanthine-guanine phosphoribosyltransferase (HPRT) mRNA levels were reduced (Figure 3). Western blot analysis showed less HPRT protein in tissue lysates that were available from two of the RE specimens compared with one of the CD specimens (Figure 4). The other housekeeping genes did not differ between the RE and CD groups [see Additional file 1: Table S1]).

Four of the seven genes with higher expression in the RE specimens, that is, the cytokine IFN-γ, and the chemokines, CCL5/RANTES (regulated and normal T cell expressed and secreted), CXCL9/MIG (monokine induced by IFN-γ), CXCL10/IP-10 (IFN-γ-induced protein 10), play a role in T cell-mediated immunity (see Discussion). Relative expression levels of these genes in the RE cohort were highly positively correlated with each other (Table 3); the lower amounts of these transcripts in the CD cohort were not correlated (Table 4). The remaining genes encode two other chemokines, CCL22/MDC (macrophage derived chemokine) and CCL23/MIP-3 (macrophage inflammatory protein 3), and Fas ligand (FasL). FasL mRNA levels positively correlated with IFN-γ, CCL5, and CXCL9 mRNA levels (Table 3); correlation between FasL and CXCL10 did not quite reach the threshold for significance. The amounts of CCL22 and CCL23 mRNA did not correlate with expression of the other genes (Table 3).

**Table 3:** Cross-correlation matrix (Spearman rank correlation coefficients) generated by comparing gene expression levels with clinical parameters of the Rasmussen encephalitis cohort

	**CCL5**	**CCL22**	**CCL23**	**CXCL9**	**CXCL10**	**FasL**	**FLAIR**	**IFN-γ**	**T2**	**Time**	**AED**
**CCL5**	1.00	**0.66**^**a**^	0.31	**0.73**	**0.64**	**0.83**	0.12	**0.94**	0.17	**−0.62**	0.07
**CCL22**		1.00	0.06	0.54	0.54	0.22	−0.44	0.5	−0.08	−0.54	0.28
**CCL23**			1.00	0.12	−0.08	0.50	0.44	0.28	**0.64**	−0.21	0.08
**CXCL9**				1.00	**0.92**	**0.63**	0.01	**0.82**	−0.24	**−0.82**	−0.07
**CXCL10**					1.00	0.53	0.00	**0.73**	−0.33	**−0.69**	0.00
**FasL**						1.00	**0.60**	**0.87**	0.25	−0.54	−0.02
**FLAIR**							1.00	0.19	0.31	−0.09	−0.19
**IFN-γ**								1.00	0.07	**−0.64**	−0.09
**T2**									1.00	0.27	0.18
**Time**^**b**^										1.00	0.12
**AED**											1.00

^a^Statistically significant correlations are in bold type (*P* < 0.05). ^b^Time from seizure onset to surgery.

AED, antiepileptic drugs.

**Table 4:** Cross-correlation matrix (Spearman rank correlation coefficients) generated by comparing gene expression levels with clinical parameters of the cortical dysplasia cohort

	**CCL5**	**CCL22**	**CCL23**	**CXCL9**	**CXCL10**	**FasL**	**FLAIR**	**IFN-γ**	**T2**	**Time**	**AED**
**CCL5**	1.00	−0.16	0.26	0.3	0.06	0.17	−0.02	0.06	−0.15	0.36	0.43
**CCL22**		1.00	−0.01	−0.02	0.06	0.08	0.04	0.03	−0.4	0.03	−0.31
**CCL23**			1.00	0.3	−0.19	0.57	0.21	0.1	0.16	0.12	0.22
**CXCL9**				1.00	0.08	0.49	0.16	−0.46	−0.1	−0.05	0.08
**CXCL10**					1.00	−0.12	−0.26	−0.2	−0.42	0.13	0.04
**FasL**						1.00	0.37	−0.15	0.39	0.23	0.56
**FLAIR**							1.00	0.18	0.41	0.57	0.25
**IFN-γ**								1.00	−0.04	**0.62**^**a**^	0.31
**T2**									1.00	0.18	0.13
**Time**^**b**^										1.00	0.51
**AED**											1.00

^a^Statistically significant correlation is in bold type (*P* < 0.05). ^b^Time from seizure onset to surgery.

AED, antiepileptic drugs.

### Inflammatory gene expression correlated with clinical parameters

**Figure 5:**
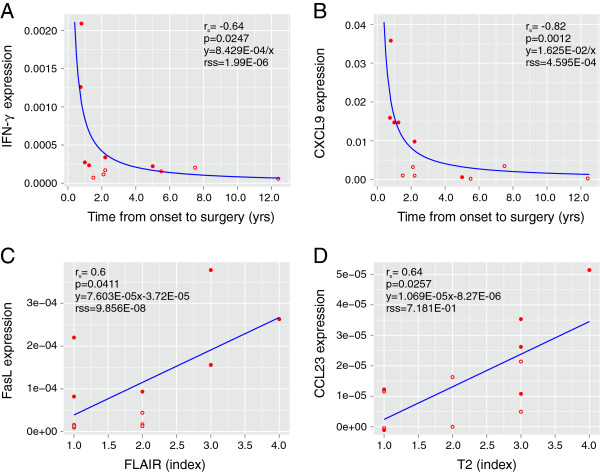
**Correlations between clinical parameters and gene expression.** Transcript levels of four of the inflammatory genes that were expressed at a significantly higher level in the Rasmussen encephalitis (RE) compared with cortical dysplasia specimens were plotted against clinical parameters. Negatively correlated parameters are best described by non-linear inverse functions (**A** and **B**), whereas linear functions can be fitted to the positively correlated parameters (**C** and **D**). Solid circles identify the six RE specimens separable by consensus clustering (Figure 2); open circles identify the remaining RE cases.

We next asked whether the expression of the seven inflammatory genes associated with RE correlated with clinical variables (Table 1). Time from seizure onset to surgery, a measure of disease progression, negatively correlated with mRNA levels for IFN-γ, CCL5, CXCL9 and CXCL10 (*P* <0.05; Table 3). By contrast, in the CD group the lower amounts of IFN-γ mRNA positively correlated with time from seizure onset to surgery (*P* <0.05; Table 4). Positive correlations were found comparing FasL mRNA and severity of inflammation, and CCL23 levels and tissue destruction as assessed by FLAIR and T2 MRIs, respectively (Table 3). As shown in Figure 5A and 5B, the data for IFN-γ and CXCL9 best fit nonlinear functions. Of the six RE cases identified by consensus clustering, five correspond to the earliest times points (filled circles in Figure 5A and 5B), suggesting that an early highly inflammatory phase distinguishes RE from CD. The FasL and CCL23 results are best described by linear functions (Figure 5C and 5D). The number of antiepileptic drugs that the RE and CD patients were receiving at the time of surgery did not significantly correlate with the expression of any of the seven inflammatory genes (Tables 3 and 4).

### T cell infiltration in Rasmussen encephalitis brain correlated with levels of inflammatory genes

**Figure 6:**
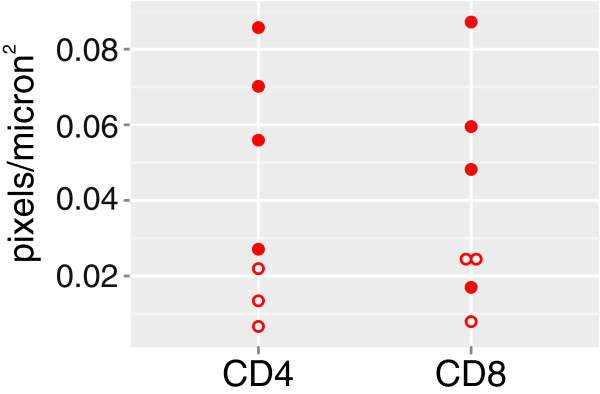
**Relative numbers of CD4**^**+ **^**and CD8**^**+ **^**T cells in sections taken from 7 of the 12 Rasmussen encephalitis (RE) cases.** Digitized images from serial sections immunostained with anti-CD4 and anti-CD8 were quantified, and indicate higher numbers of both CD4^+^ and CD8^+^ T cells in three of four RE cases identified by consensus clustering (filled circles) compared to three RE cases that were not distinguished from cortical dysplasia (open circles).

**Figure 7:**
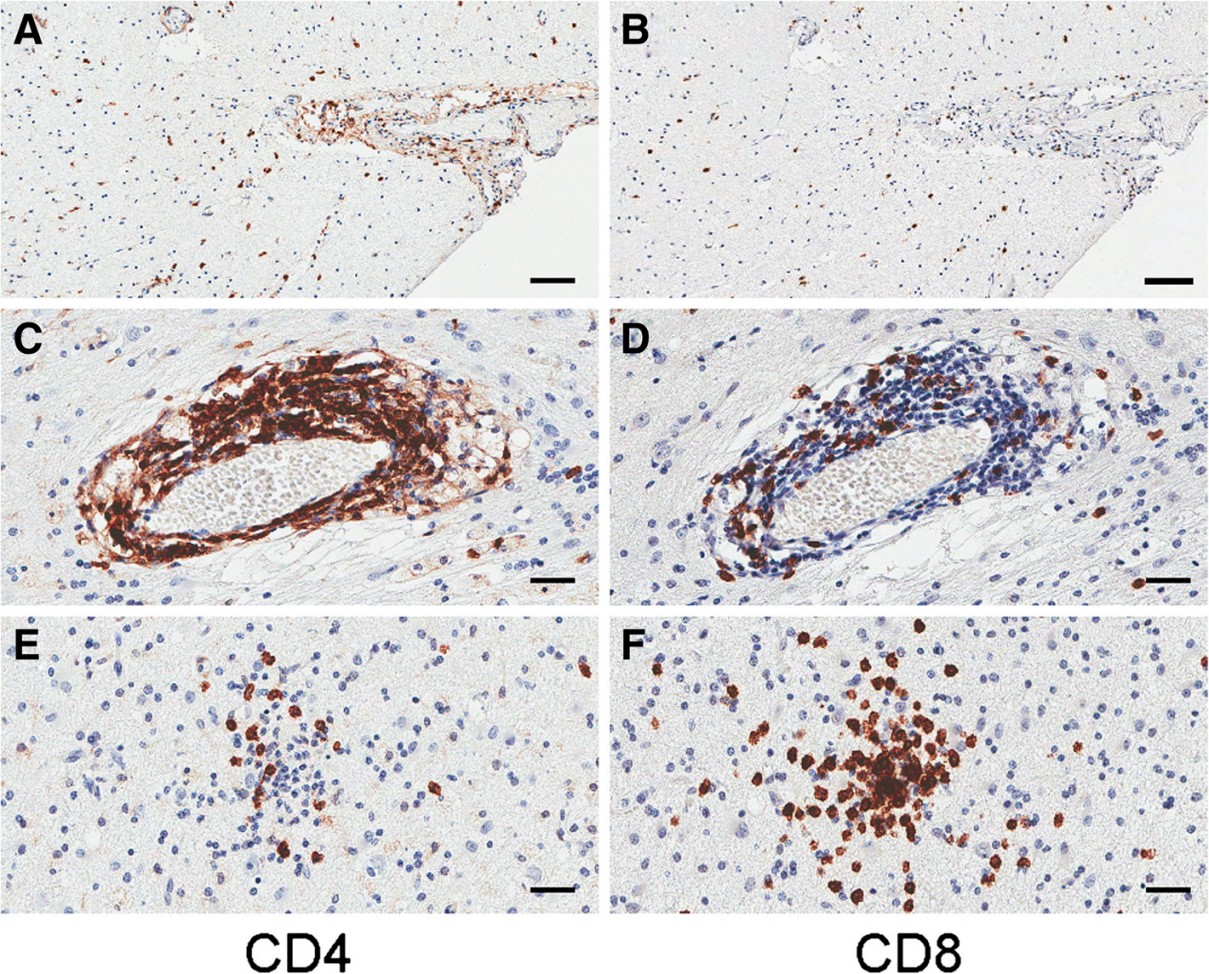
**Photomicrographs showing CD4**^**+ **^**and CD8**^**+ **^**immunoreactivity in Rasmussen encephalitis (RE) specimens.** Adjacent sections from specimens expressing high chemokine mRNA levels show a greater concentration of CD4^+^ T cells than CD8^+^ T cells within leptomeninges (**A** and **B**) and perivascular spaces (**C** and **D**), and fewer CD4^+^ T cells (**E**) within clusters of CD8^+^ T cells in brain parenchyma (**F**). Images are taken from specimens RE2 (**A** and **B**), and RE10 (**C-F**). Scale bars correspond to 50 microns (**A** and **B**), and 25 microns (**C-F**).

Based on our findings we predicted that a more pronounced T cell infiltration would be evident in tissue specimens with higher expression of IFN-γ mRNA. Sections from blocks that were available from seven of the 12 RE specimens were immunostained for CD4 and CD8 expression (four from the group of six with the highest levels of IFN-γ mRNA: RE1, RE2, RE5, and RE10, and three from the remaining specimens: RE3, RE4, and RE8). Quantitation of the strongly immunoreactive cells in digitized images of the stained sections revealed that there were more CD4^+^ and CD8^+^ T cells in sections from three out of the four cases with higher IFN-γ and related chemokine mRNA expression (Figure 6; filled circles). CD4^+^ T cells were associated with clusters of CD8^+^ T cells, but were mainly concentrated in leptomeninges and perivascular (Virchow-Robin) spaces, which contained fewer CD8^+^ T cells (Figure 7).

## Discussion

We used a qPCR array to measure inflammatory gene expression in 12 RE specimens and 12 CD specimens, and then correlated the expression of genes that differed between the two groups with clinical parameters. Selection of the CD specimens from the UCLA Pediatric Surgery Program’s tissue bank was based on choosing patients whose ages at surgery were approximately the same as the much rarer RE cases that took approximately ten years to accrue. There were no other exclusion criteria. The array included genes encoding pro-inflammatory T helper 1 (Th1) and anti-inflammatory T helper 2 (Th2) cell cytokines, chemokines, Toll-like receptors and other factors involved in downstream signaling. The expression levels of only eight transcripts significantly differed between the RE and CD specimens. With the exception of HPRT mRNA, the transcripts were expressed at higher levels in the RE specimens. Reduced HPRT, a housekeeping gene on the array, was unexpected and may reflect disease-associated atrophy. Alternatively, a reduced level of HPRT in brain tissue may be significant since a decrease in this enzyme would be expected to affect purine metabolism, with possible effects on brain function [36]. Analysis of HPRT protein levels in more RE samples will be necessary to establish the significance of this finding to the disease process.

In RE there is clear histopathologic evidence for the involvement of CD8^+^ T cells in the disease process [6-8,23]. In agreement with these data, four of the seven genes whose mRNA levels were higher in RE compared with CD cases encode proteins involved in Th1-driven immune responses, namely IFN-γ, CCL5, CXCL9 and CXCL10. Activated CD8^+^ cytotoxic T cells (Tc1) and CD4^+^ Th1 cells produce IFN- γ [37,38], thus infiltrating CD8^+^ T cells in neuropil and CD4^+^ T cells in Virchow-Robin spaces and leptomeninges (Figure 7) could be sources of IFN-γ transcripts in the RE brain specimens. Although the immunopathology in RE appears to be driven by Tc1 cells, CD4^+^ cells, in the perivascular space may play a role in sustaining Tc1 activity [39-41]. Natural killer (NK) cells, NKT cells, and γδ T cells produce IFN-γ [37,42], which may also account for the IFN-γ transcripts in RE tissue. To date, we and others have found no evidence for significant numbers of NK cells in RE brain tissue [7]. However, we have recently identified γδ T cells in brain infiltrating lymphocytes isolated from fresh RE brain tissue (unpublished data).

IFN-γ can induce major histocompatibility complex (MHC) class I molecules on the surface of neurons, rendering them vulnerable to attack by autoantigen-sensitized MHC class I-restricted Tc1 cells [43]. Further, IFN-γ has been shown to induce bursting of hippocampal pyramidal neurons *in vitro*[44], providing a possible link between T cells and epileptogenesis.

It has been reported that IFN-γ induces the production of CXCL9 by microglia and CXCL10 by microglia and astrocytes [45], and promotes IL-1-induced synthesis of CCL5 by astrocytes [46]. The positive correlation between the relative amounts of CXCL9, CXCL10, CCL5 and IFN-γ mRNA that we observed among the RE specimens is consistent with these reports. All three chemokines have been implicated in attracting Th1, Tc1, γδ T cells and NK cells to sites of inflammation [47]. The presence of CXCR3, the receptor for CXCL9 and CXCL10 [48], and CCR5 (CCL5 receptor) on infiltrating T cells in brain sections from a single RE case has been documented [49]. Other single patient studies have provided evidence for the expression of CCL5 and CXCL10 in RE brain tissue [50,51].

Quantitative PCR allowed correlations to be made between the amount of inflammatory gene transcripts and clinical variables. Notably, much higher levels of IFN-γ and CXCL9 mRNAs were detected in specimens from patients that had undergone surgery within shorter times from disease onset compared to later times. This suggests that there is a pronounced Th1 immune response in the early phase of the disease that declines after 1 to 2 years. The observation of large numbers of CD4^+^ and CD8^+^ lymphocytes in sections from specimens in which high levels of IFN-γ mRNA were detected is consonant with an initial Th1 polarized response. In support of these data, it was previously reported that cerebrospinal fluid levels of IFN-γ were higher in the earlier stages of RE [52].

A role for the Fas/FasL-mediated cell death pathway in RE is indicated from the qPCR data. Based on the observation of granzyme B immunoreactivity in T cells in close apposition to neurons and astrocytes, it has been suggested that MHC class I-restricted killing occurs by the perforin lytic pathway [6,9]. However MHC class I-restricted killing of neurons can also occur by Fas ligand-induced apoptosis [53]. On the other hand, the detection of FasL transcripts in the present study could be explained by activation-induced cell death of activated T cells in the brain [54]. In experimental autoimmune encephalitis, astrocytes expressing FasL have been implicated in T cell homeostasis [55].

In contrast to the chemokines associated with a Th1 response, CCL22, which binds CCR4 [56], is associated with a Th2 polarized response, which can facilitate B cell activation [38]. The finding of higher levels of this chemokine in RE brain tissue is therefore consistent with the presence of circulating antibodies to neuronal proteins in some RE patients [10,11,14-16]. Unlike the Th1 cytokines, CCL22 expression did not strongly correlate with time from disease onset to surgery, supporting the notion that a B cell response may be a secondary consequence of tissue destruction mediated by Tc1 cells [17]. CCL22 may also be involved in recruiting immunosuppressive T regulatory cells into the brain to modulate the Tc1 response [57].

A positive correlation was observed between the extent of tissue destruction as measured by MRI and the level of CCL23 mRNA. CCL23 can act as a chemoattractant for monocytes [58]. A role for monocytes in RE pathogenesis is therefore possible as is the case in viral encephalitis [59]. Since monocytes also produce vascular endothelial growth factor [60], this may explain the vascular changes that have been observed in resected RE but not CD brain tissue [61].

## Conclusions

Local inflammation of the brain is seen in both RE and CD, however quantitative differences in a small set of genes associated with inflammation and autoimmunity can clearly distinguish early phase RE cases from CD. In RE brain tissue, the levels of IFN-γ, CXCL9, CXCL10 and CCL5 mRNA were inversely related to the length of time between disease onset and surgery, suggesting a pronounced Th1 response in the early symptomatic phase of the disease. Differences in the number of CD4^+^ and CD8^+^ T cells in brain sections from the same cases supported this conclusion. An unresolved Th1 response can lead to autoimmunity [62], and this may explain the persistent inflammation, presence of circulating antibodies to neuronal proteins, and tissue destruction in RE. One outcome of the present study is the suggestion that blockade of chemokines could constitute a new therapeutic avenue for controlling RE disease progression [63].

## Abbreviations

CCL5/RANTES: regulated and normal T cell expressed and secreted; CCL22/MDC: macrophage derived chemokine; CCL23/MIP-3: macrophage inflammatory protein 3; CXCL9/MIG: monokine induced by IFN-γ; CXCL10/IP-10: IFN-γ-induced protein 10; CD: cortical dysplasia; FasL: Fas ligand; FLAIR: fluid attenuated inversion recovery; GAPDH: glyceraldehyde 3-phosphate dehydrogenase; HPRT: hypoxanthine-guanine phosphoribosyltransferase; IFN-γ: interferon-gamma; RE: Rasmussen encephalitis.

## Competing interests

The authors declare that they have no competing interests.

## Authors’ contributions

GCO designed study, performed qPCR, analyzed the data and drafted the manuscript; MNH performed immunocytochemistry; JWC performed western blot analysis; DLMcA carried out statistical analysis; MJH assisted with data interpretation and helped draft the manuscript; HVV provided tissue sections and helped draft the manuscript, GWM provided surgical specimens, evaluated MRI scans, and helped draft the manuscript, CAK, provided project oversight and helped draft the manuscript. All authors read and approved the final manuscript.

## Supplementary Material

Additional file 1: Table S1 The Harrell–Davis distribution-free quantile estimator method applied to a pairwise comparison of the expression of each gene. Click here for file
