# Supplementary material for: Differential expression of interferon-γ and chemokine genes distinguishes Rasmussen encephalitis from cortical dysplasia and provides evidence for an early Th1 immune response
Source: J Neuroinflammation. 2013 May 2;10:56. doi: 10.1186/1742-2094-10-56 (PMC3657540; doi:10.1186/1742-2094-10-56)
Supplement: Additional file 1: Table S1 — The Harrell–Davis distribution-free quantile estimator method applied to a pairwise comparison of the expression of each gene. [file 1742-2094-10-56-S1.docx]

Additional file 1:

Table S1: The Harrell–Davis distribution-free quantile estimator method* applied to a pairwise comparison of the expression of each gene.

| **Gene** | **1^st^ quantile** | **2^nd^ quantile** | **3^rd^ quantile** | **4^th^ quantile** | **5^th^ quantile** | **6^th^ quantile** | **7^th^ quantile** | **8^th^ quantile** | **9^th^ quantile** |
| --- | --- | --- | --- | --- | --- | --- | --- | --- | --- |
|  | p value | p value | p value | p value | p value | p value | p value | p value | p value |
| BCL6 | 0.656 | 0.852 | 0.792 | 0.696 | 0.639 | 0.674 | 0.728 | 0.735 | 0.522 |
| C3 | 0.296 | 0.823 | 0.773 | 0.747 | 0.842 | 0.815 | 0.456 | 0.232 | 0.081 |
| C3AR1 | 0.061 | 0.213 | 0.376 | 0.692 | 0.977 | 0.608 | 0.377 | 0.269 | 0.225 |
| C4A | 0.016 | 0.045 | 0.083 | 0.11 | 0.171 | 0.245 | 0.24 | 0.2 | 0.184 |
| CCL11 | 0.002 | 0.005 | 0.013 | 0.031 | 0.051 | 0.061 | 0.065 | 0.048 | 0.013 |
| CCL13 | 0.039 | 0.073 | 0.095 | 0.088 | 0.09 | 0.066 | 0.073 | 0.076 | 0.058 |
| CCL17 | 0.217 | 0.389 | 0.56 | 0.733 | 0.819 | 0.652 | 0.458 | 0.325 | 0.171 |
| CCL19 | 0.551 | 0.466 | 0.482 | 0.39 | 0.348 | 0.308 | 0.266 | 0.261 | 0.22 |
| CCL2 | 0.218 | 0.333 | 0.389 | 0.407 | 0.588 | 0.834 | 0.869 | 0.398 | 0.234 |
| CCL21 | 0.728 | 0.535 | 0.526 | 0.394 | 0.417 | 0.413 | 0.263 | 0.144 | 0.064 |
| **CCL22** | **0.002** | **0.011** | **0.02** | **0.029** | **0.036** | **0.031** | **0.007** | **0.004** | **0.004** |
| **CCL23** | **0.043** | **0.025** | **0.025** | **0.019** | **0.012** | **0.004** | **0.003** | **0.001** | **0.001** |
| CCL3 | 0.44 | 0.673 | 0.687 | 0.677 | 0.702 | 0.684 | 0.649 | 0.454 | 0.359 |
| CCL4 | 0.499 | 0.988 | 0.711 | 0.51 | 0.415 | 0.272 | 0.177 | 0.101 | 0.148 |
| **CCL5** | **0.006** | **0.014** | **0.022** | **0.023** | **0.014** | **0.005** | **0.004** | **0.001** | **0** |
| CCL7 | 0.85 | 0.812 | 0.929 | 0.917 | 0.888 | 0.68 | 0.392 | 0.197 | 0.193 |
| CCL8 | 0.236 | 0.272 | 0.317 | 0.499 | 0.525 | 0.552 | 0.577 | 0.666 | 0.64 |
| CCR1 | 0.705 | 0.514 | 0.295 | 0.169 | 0.111 | 0.036 | 0.011 | 0.003 | 0.002 |
| CCR2 | 0.345 | 0.733 | 0.929 | 0.991 | 0.914 | 0.676 | 0.451 | 0.267 | 0.158 |
| CCR3 | 0.893 | 0.952 | 0.958 | 0.914 | 0.894 | 0.621 | 0.413 | 0.192 | 0.093 |
| CCR4 | 0.664 | 0.608 | 0.434 | 0.317 | 0.225 | 0.172 | 0.095 | 0.031 | 0.03 |
| CCR7 | 0.557 | 0.186 | 0.08 | 0.087 | 0.134 | 0.152 | 0.187 | 0.135 | 0.049 |
| CD40 | 0.214 | 0.17 | 0.135 | 0.081 | 0.058 | 0.041 | 0.054 | 0.104 | 0.161 |
| CD40LG | 0.833 | 0.881 | 0.653 | 0.556 | 0.638 | 0.836 | 0.914 | 0.603 | 0.311 |
| CEBPB | 0.474 | 0.129 | 0.083 | 0.091 | 0.221 | 0.422 | 0.628 | 0.987 | 0.597 |
| CRP | 0.617 | 0.656 | 0.672 | 0.68 | 0.785 | 0.935 | 0.891 | 0.684 | 0.858 |
| CSF1 | 0.391 | 0.799 | 0.904 | 0.717 | 0.557 | 0.336 | 0.204 | 0.123 | 0.045 |
| CXCL1 | 0.262 | 0.627 | 0.803 | 0.885 | 0.986 | 0.708 | 0.43 | 0.143 | 0.066 |
| **CXCL10** | **0.044** | **0.032** | **0.029** | **0.02** | **0.011** | **0.007** | **0.002** | **0** | **0** |
| CXCL2 | 0.528 | 0.853 | 0.498 | 0.326 | 0.303 | 0.311 | 0.328 | 0.263 | 0.152 |
| CXCL3 | 0.856 | 0.445 | 0.384 | 0.33 | 0.34 | 0.356 | 0.303 | 0.158 | 0.087 |
| CXCL5 | 0.978 | 0.804 | 0.731 | 0.745 | 0.748 | 0.783 | 0.806 | 0.929 | 0.704 |
| CXCL6 | 0.402 | 0.845 | 0.97 | 0.894 | 0.635 | 0.357 | 0.151 | 0.04 | 0.009 |
| **CXCL9** | **0** | **0** | **0** | **0** | **0** | **0.003** | **0.002** | **0.001** | **0** |
| CXCR4 | 0.727 | 0.206 | 0.08 | 0.039 | 0.045 | 0.043 | 0.06 | 0.045 | 0.092 |
| **FASLG** | **0** | **0** | **0** | **0** | **0** | **0.001** | **0.001** | **0.001** | **0** |
| FLT3LG | 0.046 | 0.036 | 0.034 | 0.053 | 0.103 | 0.106 | 0.136 | 0.169 | 0.219 |
| FOS | 0.17 | 0.201 | 0.252 | 0.258 | 0.321 | 0.414 | 0.439 | 0.379 | 0.196 |
| HDAC4 | 0.761 | 0.904 | 0.991 | 0.978 | 0.894 | 0.607 | 0.429 | 0.261 | 0.172 |
| **IFNG** | **0.001** | **0.002** | **0** | **0.002** | **0.001** | **0** | **0** | **0.002** | **0.005** |
| IL10 | 0.503 | 0.336 | 0.291 | 0.293 | 0.492 | 0.738 | 0.994 | 0.698 | 0.506 |
| IL10RB | 0.118 | 0.197 | 0.304 | 0.344 | 0.393 | 0.567 | 0.859 | 0.806 | 0.707 |
| IL18 | 0.228 | 0.225 | 0.282 | 0.33 | 0.423 | 0.403 | 0.401 | 0.396 | 0.446 |
| IL18RAP | 0.347 | 0.76 | 0.889 | 0.766 | 0.645 | 0.664 | 0.674 | 0.484 | 0.561 |
| IL1A | 0.37 | 0.835 | 0.732 | 0.445 | 0.313 | 0.336 | 0.452 | 0.476 | 0.348 |
| IL1B | 0.07 | 0.095 | 0.119 | 0.132 | 0.162 | 0.081 | 0.025 | 0.007 | 0.008 |
| IL1R1 | 0.149 | 0.311 | 0.513 | 0.716 | 0.936 | 0.836 | 0.55 | 0.207 | 0.076 |
| IL1RAP | 0.022 | 0.074 | 0.102 | 0.115 | 0.102 | 0.142 | 0.229 | 0.484 | 0.966 |
| IL1RN | 0.262 | 0.399 | 0.473 | 0.436 | 0.424 | 0.372 | 0.25 | 0.124 | 0.118 |
| IL22 | 0.972 | 0.888 | 0.706 | 0.523 | 0.303 | 0.151 | 0.07 | 0.025 | 0.004 |
| IL22RA2 | 0.096 | 0.107 | 0.108 | 0.085 | 0.089 | 0.077 | 0.092 | 0.094 | 0.06 |
| IL23A | 0.03 | 0.081 | 0.181 | 0.384 | 0.494 | 0.502 | 0.357 | 0.187 | 0.103 |
| IL23R | 0.4945 | 0.518 | 0.497 | 0.478 | 0.4355 | 0.356 | 0.2085 | 0.1495 | 0.116 |
| IL6 | 0.683 | 0.842 | 0.85 | 0.922 | 0.656 | 0.373 | 0.198 | 0.092 | 0.064 |
| IL6R | 0.322 | 0.606 | 0.71 | 0.875 | 0.884 | 0.973 | 0.831 | 0.67 | 0.512 |
| IL8 | 0.395 | 0.379 | 0.333 | 0.252 | 0.112 | 0.06 | 0.029 | 0.011 | 0.004 |
| CXCR1 | 0.547 | 0.77 | 0.971 | 0.803 | 0.777 | 0.851 | 0.794 | 0.412 | 0.114 |
| CXCR2 | 0.479 | 0.682 | 0.815 | 0.847 | 0.783 | 0.72 | 0.547 | 0.354 | 0.283 |
| IL9 | 0.557 | 0.897 | 0.853 | 0.556 | 0.306 | 0.163 | 0.096 | 0.075 | 0.064 |
| ITGB2 | 0.317 | 0.567 | 0.735 | 0.769 | 0.662 | 0.422 | 0.206 | 0.114 | 0.06 |
| KNG1 | 0.849 | 0.774 | 0.769 | 0.88 | 0.965 | 0.858 | 0.824 | 0.82 | 0.671 |
| LTA | 0.562 | 0.768 | 0.947 | 0.878 | 0.822 | 0.911 | 0.942 | 0.804 | 0.936 |
| LTB | 0.713 | 0.629 | 0.344 | 0.163 | 0.104 | 0.109 | 0.18 | 0.277 | 0.355 |
| LY96 | 0.453 | 0.571 | 0.883 | 0.791 | 0.526 | 0.389 | 0.301 | 0.257 | 0.17 |
| MYD88 | 0.983 | 0.896 | 0.888 | 0.737 | 0.617 | 0.418 | 0.255 | 0.111 | 0.037 |
| NFATC3 | 0.023 | 0.069 | 0.17 | 0.344 | 0.632 | 0.916 | 0.916 | 0.66 | 0.3 |
| NFKB1 | 0.812 | 0.812 | 0.674 | 0.62 | 0.634 | 0.775 | 0.998 | 0.678 | 0.324 |
| NOS2 | 0.91 | 0.669 | 0.685 | 0.869 | 0.918 | 0.731 | 0.733 | 0.834 | 0.875 |
| NR3C1 | 0.003 | 0.021 | 0.073 | 0.204 | 0.406 | 0.676 | 0.799 | 0.744 | 0.585 |
| RIPK2 | 0.021 | 0.023 | 0.05 | 0.09 | 0.142 | 0.319 | 0.531 | 0.759 | 0.856 |
| TIRAP | 0.376 | 0.779 | 0.922 | 0.823 | 0.618 | 0.425 | 0.266 | 0.17 | 0.14 |
| TLR1 | 0.747 | 0.832 | 0.99 | 0.807 | 0.752 | 0.73 | 0.807 | 0.691 | 0.523 |
| TLR2 | 0.241 | 0.176 | 0.156 | 0.107 | 0.132 | 0.138 | 0.169 | 0.144 | 0.089 |
| TLR3 | 0.916 | 0.812 | 0.673 | 0.47 | 0.322 | 0.173 | 0.079 | 0.027 | 0.002 |
| TLR4 | 0.899 | 0.415 | 0.273 | 0.202 | 0.162 | 0.178 | 0.252 | 0.27 | 0.269 |
| TLR5 | 0.875 | 0.714 | 0.453 | 0.358 | 0.383 | 0.411 | 0.528 | 0.499 | 0.481 |
| TLR6 | 0.173 | 0.248 | 0.271 | 0.209 | 0.171 | 0.099 | 0.094 | 0.048 | 0.031 |
| TLR7 | 0.621 | 0.889 | 0.901 | 0.68 | 0.511 | 0.272 | 0.125 | 0.054 | 0.044 |
| TNF | 0.783 | 0.925 | 0.968 | 0.977 | 0.892 | 0.806 | 0.58 | 0.585 | 0.68 |
| TNFSF14 | 0.844 | 0.467 | 0.166 | 0.043 | 0.006 | 0.003 | 0.001 | 0 | 0 |
| TOLLIP | 0.024 | 0.062 | 0.157 | 0.352 | 0.596 | 0.879 | 0.76 | 0.571 | 0.432 |
| B2M | 0.487 | 0.259 | 0.119 | 0.054 | 0.046 | 0.013 | 0.006 | 0.004 | 0.001 |
| **HPRT1** | **0** | **0** | **0.004** | **0.003** | **0.004** | **0.003** | **0.001** | **0.001** | **0.001** |
| RPL13A | 0.204 | 0.451 | 0.774 | 0.962 | 0.785 | 0.667 | 0.585 | 0.529 | 0.489 |
| GAPDH | 0.007 | 0.02 | 0.032 | 0.019 | 0.019 | 0.035 | 0.069 | 0.195 | 0.439 |

*The method compares the expected value at each quantile and generates a p value.

Statistically significant differences in expression level across all quantiles are indicated in bold face.
